# End-to-End Deep One-Class Learning for Anomaly Detection in UAV Video Stream

**DOI:** 10.3390/jimaging7050090

**Published:** 2021-05-19

**Authors:** Slim Hamdi, Samir Bouindour, Hichem Snoussi, Tian Wang, Mohamed Abid

**Affiliations:** 1 ICD-LM2S, CNRS, University of Technology of Troyes, 10000 Troyes, France; samir.bouindour@yahoo.fr (S.B.); hichem.snoussi@utt.fr (H.S.); 2 CES Laboratory, ENIS National Engineering School, University of Sfax, Sfax 3038, Tunisia; mohamed.abid_ces@yahoo.fr; 3 School of Automation Science and Electrical Engineering, Beihang University, Beijing 100191, China; wangtian@buaa.edu.cn

**Keywords:** anomaly detection, UAV videos, deep one-class

## Abstract

In recent years, the use of drones for surveillance tasks has been on the rise worldwide. However, in the context of anomaly detection, only normal events are available for the learning process. Therefore, the implementation of a generative learning method in an unsupervised mode to solve this problem becomes fundamental. In this context, we propose a new end-to-end architecture capable of generating optical flow images from original UAV images and extracting compact spatio-temporal characteristics for anomaly detection purposes. It is designed with a custom loss function as a sum of three terms, the reconstruction loss ($R_{l}$), the generation loss ($G_{l}$) and the compactness loss ($C_{l}$) to ensure an efficient classification of the “deep-one” class. In addition, we propose to minimize the effect of UAV motion in video processing by applying background subtraction on optical flow images. We tested our method on very complex datasets called the mini-drone video dataset, and obtained results surpassing existing techniques’ performances with an AUC of 85.3.

## 1. Introduction

The use of drones is booming around the world with a large variety of potential applications: wireless acoustic networking for amateur drone surveillance [1], updating of UAV networking using the software-defined radios (SDR) and software-defined networking (SDN) [2], the multi-agent reinforcement learning (MARL) framework [3] and malicious Wi-Fi hotspots detection [4]. In particular, the use of the UAV camera has become very important in the field of detecting abnormal behaviour in video footage. This importance stems from the fact that not only can a UAV monitor large and dangerous areas, but it is also cost-effective and can replace an entire installation of fixed cameras [5]. Moreover, processing video sequences from UAV for anomaly detection is a complex task compared to its counterpart with fixed cameras for two reasons: (a) Lack of video datasets from UAV in real conditions, and (b) dynamic, variable brightness and large-scale backgrounds. A video-drone protection system is a closed-circuit television CCTV system that describes a whole range of video surveillance technologies. Many factors can significantly reduce the effectiveness of CCTV systems, such as fatigue and lassitude caused by prolonged viewing of many surveillance videos. A possible solution to this problem would be the use of intelligent video surveillance systems. These systems must be capable of analysing and modelling the normal behaviour of a monitored scene and detecting any abnormal behaviour that could represent a security risk. In recent years, considerable technological advances in the fields of machine learning and computer vision have made it possible to process CCTV systems. Some of these are classics of machine learning: image classification [6], facial recognition [7], human pose estimation [8], natural language processing [9], automatic voice recognition [10], and even more atypical tasks; machine translation systems [11], lip reading [12] and automatic software code generation [13]. Moreover, Deep Learning (DL) is a sub-domain of Machine Learning (ML), it aims to learn high-level abstractions in data using multi-level architectures. These different levels are obtained by stacking several non-linear transformation modules. Each module transforms the data at a different level until a suitable representation is obtained to perform the target task. Deep learning has made it possible to go beyond the traditional model in certain application cases and to design efficient pattern recognition systems without in-depth expertise on the target elements. In fact, the most effective deep-learning methods are based on supervised learning, using large, labelled databases containing samples from different classes. To take advantage of these learning materials in an intelligent monitoring system, a large amount of training data representative of normal and abnormal events is required. Abnormal events are the rare events that does not appear redundantly at the scene. Thus, there are many barriers to the creation of such databases—for example, we can cite the following:

- The contextual aspect of the event. Indeed, an event is closely linked to its context, an abnormal event in one scene can be normal in another. This point makes it almost impossible to design common databases that can be used uniformly for different scenes.
- Risks and variability to reproduce some abnormal events make it impossible to identify and generate enough training samples.

Abnormal video events have been called by many names in the literature, such as abnormality, irregular behaviour, unusual behaviour, or abnormal behaviour. These different names will be used alternately without worrying about technical inconsistency. The detection of abnormal video events is also characterised by a variety of strategies for processing training data. The first approach is to carry out the training only on normal data and to consider any type of event outside the training phase as abnormal. Another approach, in contrast to the first, is to use only abnormal events for training [14]. This approach can be effective in identifying a certain type of abnormal events, but presents a high risk of missing abnormal events that are different from those that have been trained. Another approach is based on the use of data labelled in two different classes, normal and abnormal [15]. Other work uses more advanced classified and labelled data where each class represents a specific type of event [13]. Approaches that use abnormal events as learning data often have limitations. Some abnormal events are impossible to reproduce. The variability of abnormal events greatly complicates the learning task and can have a negative effect on modelling. Other approaches are based on clustering methods with the usage of unlabelled databases containing both normal and abnormal data [16]. It is assumed that normal events are those that occur frequently, and abnormal events are those that occur rarely. The advantage of this approach is that it does not require any labelling of training data, but its effectiveness is compromised by the assumption that all rare events are abnormal because, obviously, a rare event is not necessarily abnormal. Despite the different strategies for training data on the detection of abnormal events [15,17,18,19], the first approach of using only normal data during training has become the norm. In our work, we adopt this approach and we propose a new architecture capable of detecting abnormal event by training only with normal samples. The rest of the paper is organized as follows: Section 2 briefly reviews related literature of this research field. Section 3 introduces the proposed method. Experimental results are shown and discussed in Section 4. Section 5 concludes this paper and addresses some potential future studies.

## 2. Related Work

For many years, the development of a pattern recognition system based on the traditional model required expertise and in-depth knowledge to extract from the raw data appropriate representations that could be used to detect, identify or classify items among the input data. These methods require a priori knowledge to construct a feature extractor adapted to the targeted events and the scene being monitored. These constraints have led to the emergence of abnormal event detection methods based on learning representations and, more precisely, on deep learning. Representation learning or feature learning is a set of techniques allowing to automate the feature extraction step. These methods make it possible to define, by learning, the appropriate transformations to be applied to the input data in order to obtain representations to perform a targeted task, such as the recognition of an action, the classification of an image, the estimation of a human pose, semantic segmentation, and so forth [6,9,20,21].

### 2.1. Transfer Learning

The CNN is a type of artificial neural network inspired from the animal visual cortex. It consists of several layers that process data in a hierarchical pattern. It has been shown that a CNN trained to perform a target task can provide generic and robust functionality that can be used to perform another computer vision task different from the one for which it has been specifically trained. In [22], representations extracted with OverFeat, a CNN trained solely in object classification, are exploited by a linear SVM or Euclidean standard for different tasks (scene classification, detailed classification, attribute detection, visual instance retrieval). The results provide tangible evidence of the CNN’s ability to provide generic and robust functionalities that can be used for different computer vision tasks. This principle has been applied in many works on abnormal event detection. In [23], a 2D CNN pre-formed from image classification databases is modified to extract representations of different regions from input images. An OC-SVM is then used to detect which of these regions have abnormal events. In [24], a pre-formed CNN is combined with a scattered self-coder that can be formed to provide a two-level feature extractor. At the output of the CNN, a first Gaussian classifier is used to classify regions of the image as normal, abnormal, or suspect. Representations of suspect regions are then transformed by the auto-coder to obtain more discriminating representations.

Methods based on transfer learning do not require a labelled database for feature extraction, and their results in terms of detection and localisation are very promising. However, the dependence of these methods on pre-trained models imposes a certain rigidity which considerably reduces their prospects for potential improvements. This drawback has originated the emergence of approaches based on generative and deep one-class models.

### 2.2. Generative Models

In recent years, the use of Generative Adversarial Networks (GANs) in machine learning has increased considerably. GAN is an unsupervised learning algorithm proposed for the first time by [25]. It consists of two sub-networks, a generator and a competing discriminator. During the learning phase, the generator tries to generate convincing data to deceive the discriminator which, in turn, tries to detect whether the generated samples are real (regular) or fakes (irregular). In [18], spatio-temporal adversary networks (STAN) was proposed to meet the challenge of video anomaly detection. It is composed of two sub-networks, a generator composed of convolution layers, ConvLSTM [26] and deconvolution layers and a discriminator composed of 3D convolution layers. The detection of abnormal events can be done directly by the discriminator or generator. However, the best results in [18] were obtained by combining the decisions of the two networks. The author of [27] also proposed the use of GANs for the detection of abnormal events. A thresholding of the generation error of the two GANs is used in order to identify the image regions containing the abnormal events. The first GAN is trained to generate optical flow representations from images, and the second GAN is trained to generate images from optical flow representations. However, the error between the generated images and the real images is not sufficient to obtain convincing results.

### 2.3. One-Class Models

Abnormal event detection approaches based on reconstructive, predictive or generative models are generally based on the assumption that a model formed on normal images will not be able to reconstruct, predict or generate abnormal images. Therefore, a threshold of reconstruction, prediction or degeneration error is often used to detect abnormal events. However, in the case of video events, the different elements of normal and abnormal situations are often similar and it is usually their interactions or the context that defines the normality or abnormality of a situation. In this respect, recent work aimed at developing one-class networks has been proposed. The ref. [28] proposes Deep One-Class (DOC), a convolutional neural network that can be trained end-to-end, using only one-class learning examples. The network is obtained by replacing the softmax usually used in CNNs with an OC-SVM. Moreover, The authors define an objective function that allows the formation of not only the OC-SVM layer, but also of all the layers of the network that can be formed. In this way, the network is optimised to extract compact representations and define the appropriate hyperplane to isolate data representations from the target class. On the other hand, many works based on one-class neural networks have been proposed for the detection of anomalies [29,30]. These works require very little adaptation to be used in the context of detection of abnormal video events. The ref. [31] proposes the use of transfer learning for adapting pre-trained networks to perform anomaly detection. The authors assume that two important aspects, compactness and description of the extracted features, must be imperatively considered. The description provides descriptive features. However, the compactness is used in order to ensure that images of the same class are described by similar representations, so they are positioned compactly in the feature space. These two aspects can significantly contribute to a decrease in the intra-class distance and an increase in the inter-class distance. To obtain these two aspects, the authors propose two networks. After the learning, the two identical networks are capable of providing both descriptive and compact representations. These networks can be applied with a One-Class classifier to dissociate the elements of a target class from the outliers. However, these methods proposed to use extra data sets or optical flow samples for analysing motion, which make these methods depend on handcrafted features and on the quality of extra datasets. In this work, we propose to build an architecture capable of analysing motion from raw images without using extra datasets.

### 2.4. Motivation and Contributions

In recent years, state-of-the-art methods have been based principally on generative or deep one-class models to treat the problem of anomaly detection efficiently. However, no single model has been proposed before being aimed at bringing together the benefits of both models. For that reason, the originality of our work is to propose a new architecture bringing together the advantages of both generative and deep one-class models for anomaly detection purposes in a UAV video footage. Our motivation is to design this new architecture in order to achieve high performance and a minimum Equal Error Rate (EER), compared to existing methods. Moreover, for many existing methods, optical flow features are computed by a pre-processing task before starting the inference. In this work, we propose an architecture capable of generating optical flow features at the testing phase, meeting the real-time constraint. The purpose of our work is to efficiently address the problem of anomaly detection by drone cameras. This purpose is ensured by creating a new deep one-class architecture capable of compacting the features of a given class into a half-hyper sphere. This classification method can be useful for many anomaly detection problems in other domains.

The contributions of our paper are summarized as follows:

- We propose a new end-to-end unsupervised generative learning architecture for deep one-class classification in order to guarantee not only the compactness of the different characteristics of normal events (optical flow and original images), but also the ability to automatically generate optical flow images from the UAV original video during the test phase, which makes the processing chain faster for abnormal event detection. We have trained our architecture with a custom loss function as a sum of three terms, the reconstruction loss ($R_{l}$), the generation loss ($G_{l}$) and the compactness loss ($C_{l}$) to ensure an efficient classification of normal/abnormal events.
- In addition, we have applied background subtraction on the UAV optical flow to minimise the effect of camera movement, and we have tested our method on complex and hard-to-reach datasets in terms of variety of content and conditions, such as mini-video datasets.

## 3. Proposed Method

**Figure 1:**
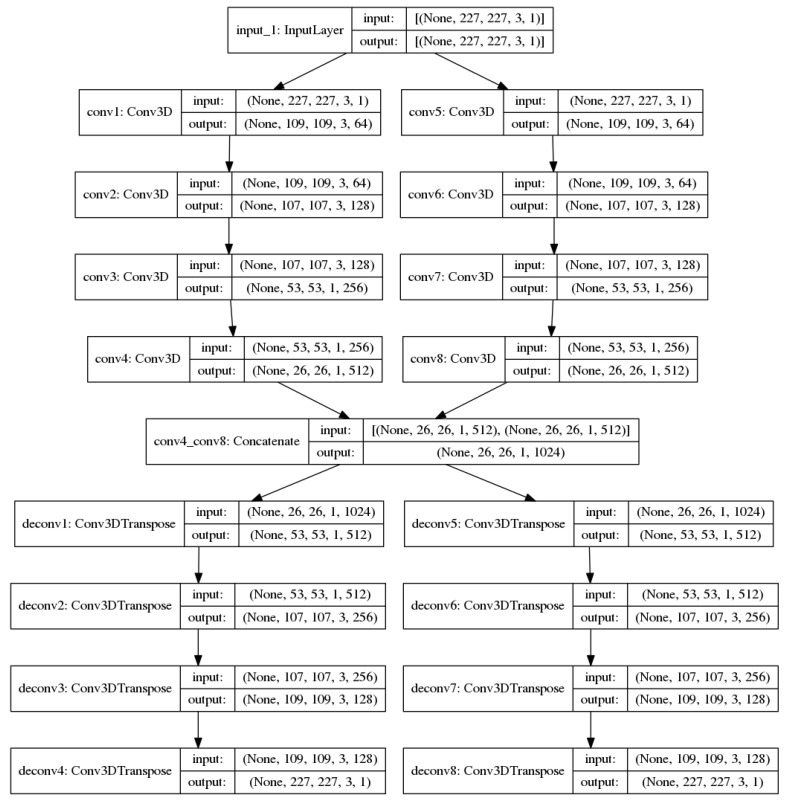
The proposed deep-learning architecture.

In this section, we propose a new end-to-end unsupervised architecture (Figure 1) for anomaly detection in UAV video footages. It is trained with only consecutive normal RGB and optical flow frames. Our architecture is capable of building new optical flow representations of a UAV video from consecutive original frames. It is based on a mix of convolution and deconvolution layers capable not only of automatically generating optical flow images, but also of extracting compact features from the original and optical flow images during the test phase. Classical computation of optical flow is then avoided and replaced by a fast and efficient convolution/deconvolution-based neural network. The proposed procedure can produce optical flow representations of abnormal samples with higher optical flow error (OFE) generation than normal samples, intuitively by decreasing the intra-class distance of the normal class during the training phase, as in the following equation:

(1) $$OFE=\frac{1}{n}\sum_{1}^{n}{\left(\phi \left(i\right)-\hat{\phi }\left(i\right)\right)}^{2},$$

where ϕ(i) is the original optical flow and $\hat{\phi }\left(i\right)$ is the generated optical flow. Thanks to this architecture, our model is able to correctly represent shapes and motion in videos. The neural network is composed of eight convolution layers: a concatenation layer, to combine the feature maps of each of the four convolution layers, and eight deconvolution layers to reconstruct the input composed of the consecutive original images and to generate the consecutive optical flow images. The concatenation layer is our bottleneck layer. We called our architecture a CNN optical flow generator because of its ability to generate optical flow samples from original images. The hyper-parameters of our architecture are provided in the following Table 1.

The Concat represents our concatenation layer; it does not need any filters or any strides as hyper-parameters, as it concatenates the outputs of the Conv4 and Conv8 layers. In the next section, we will discuss the proposed training strategy which is not limited to reconstruction error, but introduces a new concept of compactness. We will also detail the testing phase for our architecture during the inference.

### 3.1. Loss Function and Training Phase

We propose to train our architecture using only normal samples. We have used, as input volumes, three consecutive frames F= {$F_{t}$; $F_{t-1}$; $F_{t-2}$} to describe not only the shapes, but also the motion encoded in these three frames. Only in the training, the frames and their corresponding optical flow representations are extracted from the raw videos and resized to 227 × 227. We scaled the pixels values in [1, −1]. In the testing phase, we used the same scaling values as in the training to ensure the condition of real-world applications. Our architecture was trained by the Adam optimizer with a learning rate equal to 0.00001. A hyperbolic tangent is used as the activation function of each convolution and deconvolution layer to ensure the symmetry of the reconstructed and the input video volume. The original aspect of our work is to design a custom loss function (L) as the sum of three terms, as given in Equation (2): a term related to compactness $C_{l}$, a term related to generation loss $G_{l}$ and a term related to the reconstruction loss $R_{l}$. The aim of using those three loss components is to maximize the inter-class distance (between normal and abnormal samples) and to minimize the intra-class distance (between normal samples).

**Figure 2:**
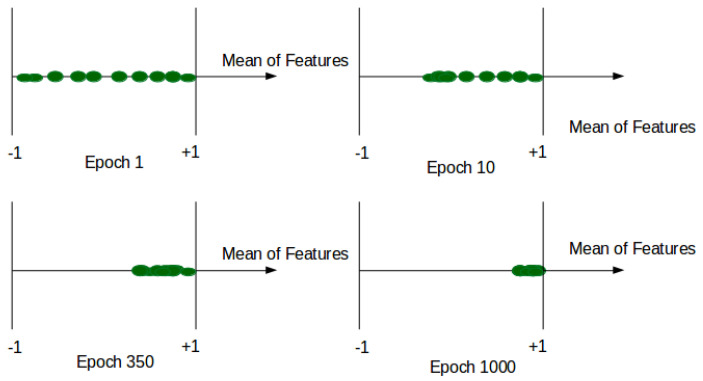
Average features during training.

The objective of the $C_{l}$ and $G_{l}$ loss terms is to obtain features capable of generalization for normal samples and also of generating optical flow images with minimum OFE. Thus, those terms aim at maximizing the inter-class distance between normal and abnormal samples. The compactness loss allows to obtain compact features (both for shape and motion) of training data by minimizing their distance to a fixed point $C_{0}$. We have fixed the point $C_{0}$ at the maximum of our data range, which is a vector of ones. The overall loss *L* is then written as:

(2) $$L=\frac{1}{n}\left(\sum_{i=1}^{n}{\left(V-\hat{V}\right)}^{2}+\sum_{i=1}^{n}{\left(W-\hat{W}\right)}^{2}\right)+\alpha \left|M(x_{i})-1\right|$$

(3) $$L=R_{l}+G_{l}+\alpha C_{l},$$

where *V* represents the volume of the original image input, $\hat{V}$ is the corresponding shape-reconstructed volume, *W* is the optical flow volume, and $\hat{W}$ is its corresponding reconstructed volume. $M(x_{i})$ is the mean value of features $x_{i}$ at each patch in the Concat layer. α is a hyper-parameter between [0, 1] of our custom loss function, and it controls the influence of the compactness of our features. In practice, we fixed α to 0.1 to ensure the scale condition of other terms of *L*. It should be noted that when α = 0, the model is trained without compactness loss and limited to reconstruction and generation loss. When $M(x_{i})$ tends to 1, the features $x_{i}$ tends to $C_{0}$. Then, we ensure that all normal features at the training are converging near the same point $C_{0}$ (see Figure 2).

### 3.2. Testing Phase

After training our architecture, we were able to obtain a model capable of extracting a robust spatio-temporal representation of each patch. Thanks to this architecture, each small region of the input video volume is represented by a 1024-vector of features capable of describing the shapes and motion contained in that region.

In the test phase, only the original images were used. Optical flow samples were generated by our architecture, which allows for fast implementation of the global detector. The compactness is used to constrain feature vectors inside a half-hypersphere (S) with centre $C_{0}$ and a small radius R, enhancing the performance of the classification procedure. For each new video volume, we extract the mean of the features $M^{\prime }\left(x_{i}\right)$ at the Concat layers and compare its distance to $C_{0}$ to the radius R:

(4) $$\left\{\begin{array}{c} Normal\;\;if\;(C_{0}-M^{\prime }\left(x_{i}\right))\pm \varepsilon \leq R\\ Abnormal\;\;if\;(C_{0}-M^{\prime }\left(x_{i}\right))\pm \varepsilon >R \end{array}\right.$$

where ε defines the insensitivity zone.

## 4. Experimental Results

We have used different datasets to evaluate the proposed detection method. The model was trained with only normal events contained in datasets, and then it was tested within different abnormal events. The used datasets are listed as follows:

- Mini-Drone Video Dataset:
  Mini-Drone Video Dataset (MDVD) [32] is a dataset filmed by a drone of type Phantom 2 in a car park. It is mainly used for events identification. It is composed of 38 videos captured in high resolution, with a duration up to 24 s each. The videos in MDVD were divided into three categories: normal, suspicious, and abnormal, and they are defined by the actions of the persons involved in the videos. The normal case is defined by several events, such as people walking, getting in their cars, or parking correctly. The abnormal cases are represented by people fighting or stealing. Finally, for suspicious cases, nothing is wrong, but people do suspicious behavior which could distract the surveillance staff. In order to use the MDVD dataset in unsupervised mode for anomaly detection, we split this dataset into: 10 videos for the training containing only normal samples, and 10 videos for the test containing both abnormal and normal events.
- USCD Ped2:
  UCSD Peds2 [33] is an anomaly detection dataset consisting of video footage of a crowded pedestrian walkway captured by a stationary camera. It contains both normal and abnormal events, like the walking movement of bikers, skaters, cyclists, and small carts. However, in the walkways, the motion of the pedestrian in an unexpected area is also considered as an anomalous event. It contains 16 training and 12 testing video samples, and provides frame-level ground truth, which helps us to evaluate the detection performance and to compare our method with other stat-of-the-art anomaly-detection methods.
- Brutal running dataset:
  We propose a new small dataset with 1000 samples (340 training samples and 660 samples for test) called the brutal running dataset captured by a Phantom 4 pro drone. The normal event consists of a girl walking outside, and the abnormal event occurs when she is running. This kind of anomaly is largely used in anomaly detection by fixed cameras.

### 4.1. Minimization of the Effect of UAV Motion on Optical Flow Images

**Figure 3:**
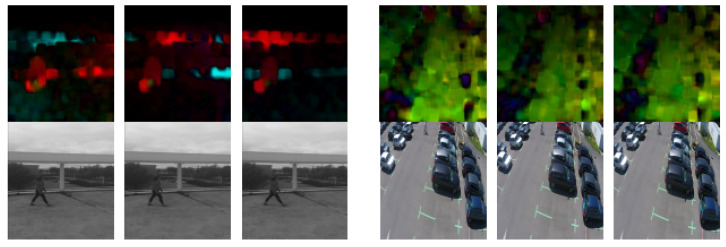
Optical flow samples of MDVD and other examples.

Optical flow is the pattern of apparent motion of objects between two consecutive frames. It is a 2D vector field, where each vector is a displacement vector showing the movement of points from the first frame to the second. For training, we used the OpenCV Gunner Farneback algorithm to extract dense optical flows. We obtained a two-channel array with optical flow vectors (u,v). The Figure 3 shows same samples of optical flow calculated by Farneback’s algorithm.

In order to denoise and minimize the effect of UAV motion on optical flow images, we propose to subtract the mean optical flow at the train and apply the same centering for the optical flow samples during testing.

**Figure 4:**
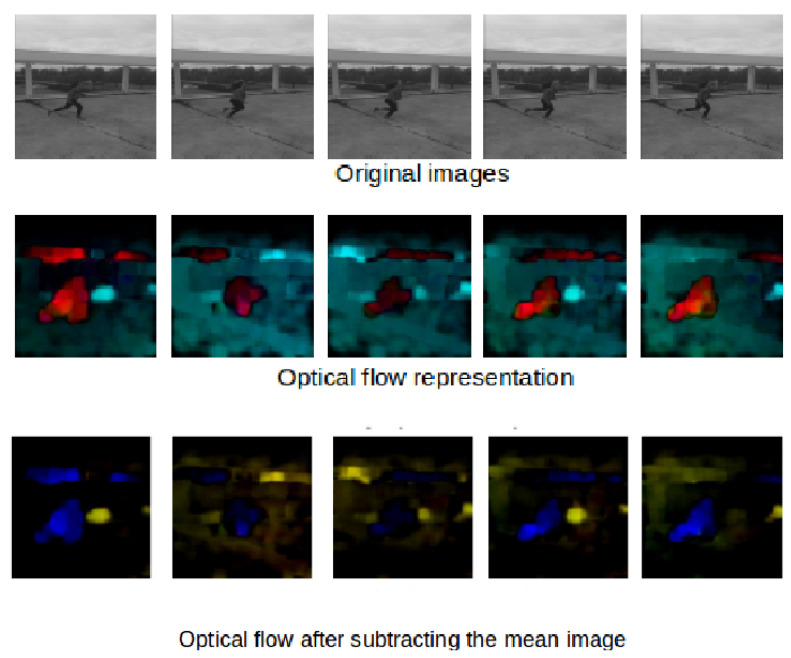
Subtraction of mean optical flow.

**Figure 5:**
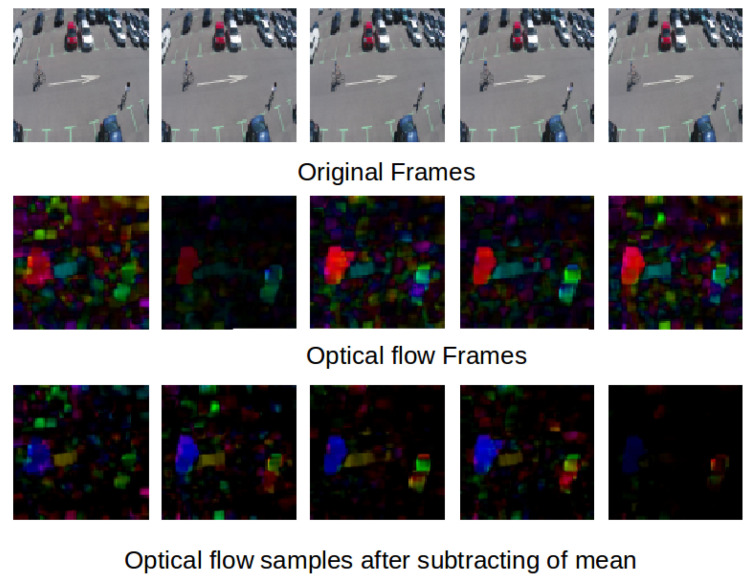
Subtraction of mean optical flow in the MDVD dataset.

Figure 4 and Figure 5 show some examples of the optical flow of the Mini drone dataset and some other examples captured in a different scene. These figures prove that subtracted mean drone motion can minimize the drone motion effect on optical flow frames which become less noisy. We have used this version of optical flow to train our architecture.

#### 4.1.1. Optical Flow Generating

**Figure 6:**
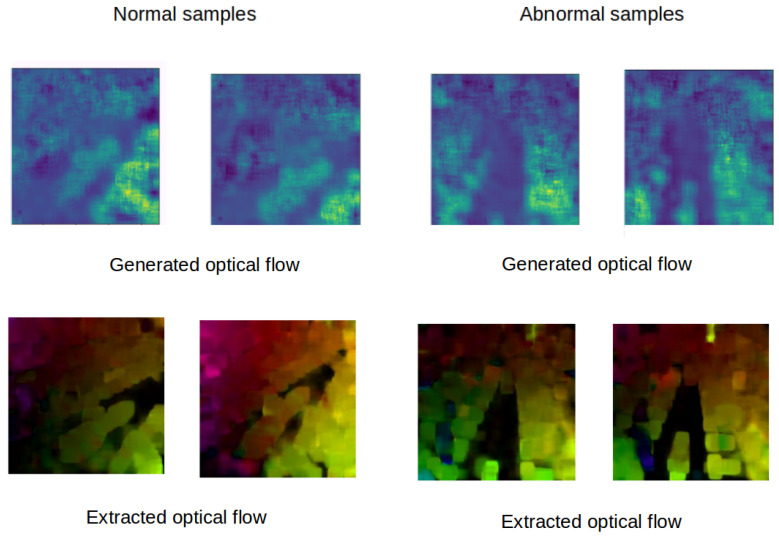
Samples of optical flow generated by our architecture.

Figure 6 shows the generated optical flow frames of both normal and abnormal samples of MDVD. It shows that our architecture can reproduce optical flow frames from original video frames. Then, at the testing phase (inference), it does not need a handcraft algorithm to extract optical flow. The proposed architecture is fed only with a raw video, directly ensuring the real-time implementation of the detection algorithm, even on constrained embedded processing units.

#### 4.1.2. Architecture Evaluation

We used Error Equal Rate (EER) and Area Under Curve ROC (AUC) as evaluation criteria. A smaller EER corresponds to better performance. As for the AUC, a bigger value corresponds to better performance. The Table 2 summarizes our results on MDVD, and a comparison was done with existing methods.

**Figure 7:**
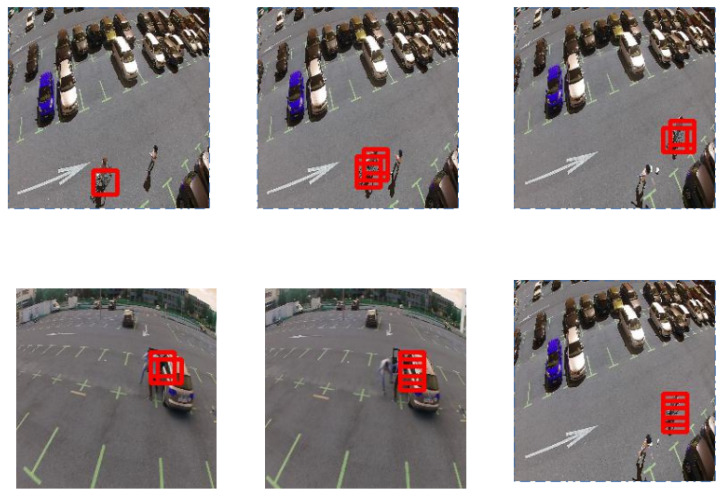
Our results on the MDVD dataset.

Figure 7 illustrates algorithm results on MDVD, and proves that our method can localize anomalies: biker and fighting events. However, when the drone motion is fast, our system can give some localisation errors, but it still can dissociate between abnormal and normal events at frame level. Despite the difference between the movements and trajectories of the drone in the training phase and the testing phase, the results corroborate the effectiveness of the proposed architecture which works properly in detecting and localizing abnormal events.

**Figure 8:**
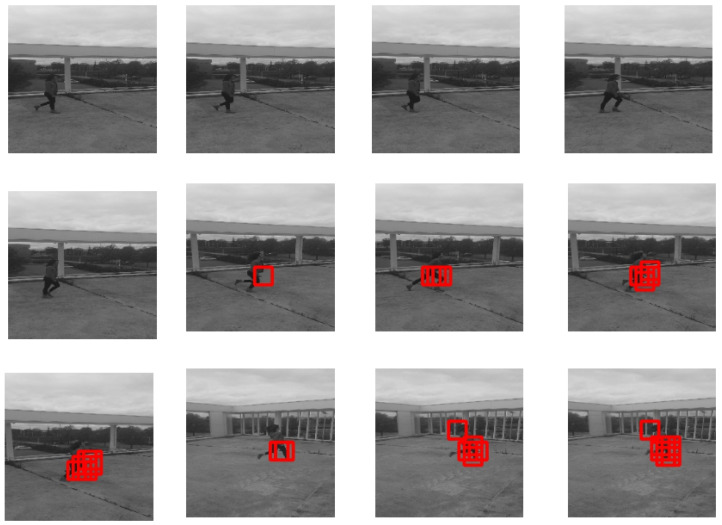
Our results on the brutal running dataset.

Figure 8 represents our results on the brutal running dataset. It shows that our method is capable of detecting abnormal brutal motion (running, in this case).

**Figure 9:**
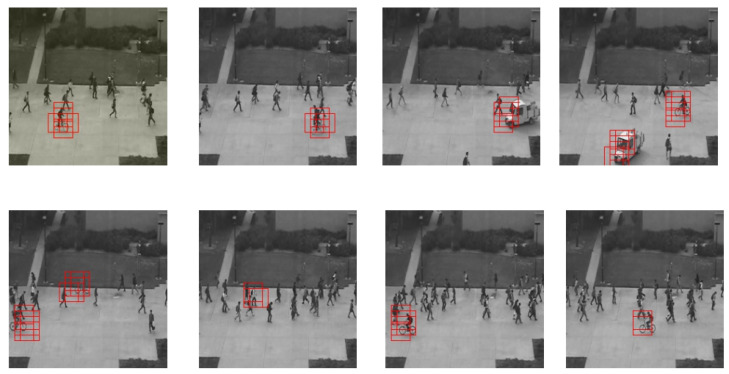
Ourresults on the Ped2 dataset.

In order to further evaluate of the proposed method, we have tested on UCSD Ped2 datasets with fixed cameras and compared our results with state-of-the-art methods. Table 3 and Figure 9 report these comparative results, showing again the effectiveness of our method in video anomaly detection.

#### 4.1.3. Compactness Evaluation

In order to evaluate the advantages of compactness loss, we trained our model with and without this loss term. Table 4 shows the obtained results from MDVD using the Mahalanobis distance (Equation (5)):

(5) $$D=\left(y_{j}-M\right)\times Q\times {\left(y_{j}-M\right)}^{\prime }\;\;Mahalanobis\;\;distance:\left\{\begin{array}{c} Normal\;\;if\;D\leq \alpha \\ Abnormal\;\;if\;D>\alpha \end{array},\right.$$

where *M* is the mean and *Q* is the inverse of the covariance matrix of the training data *X*. If the distance exceeds a threshold α, the testing vector $y_{j}$ is considered as an outlier, and the corresponding frame is labeled as abnormal. The results of Table 4 show that the compactness feature enhances the detection performances compared to the Mahalanobis classifier based on the extracted features from the Concat layer.

**Figure 10:**
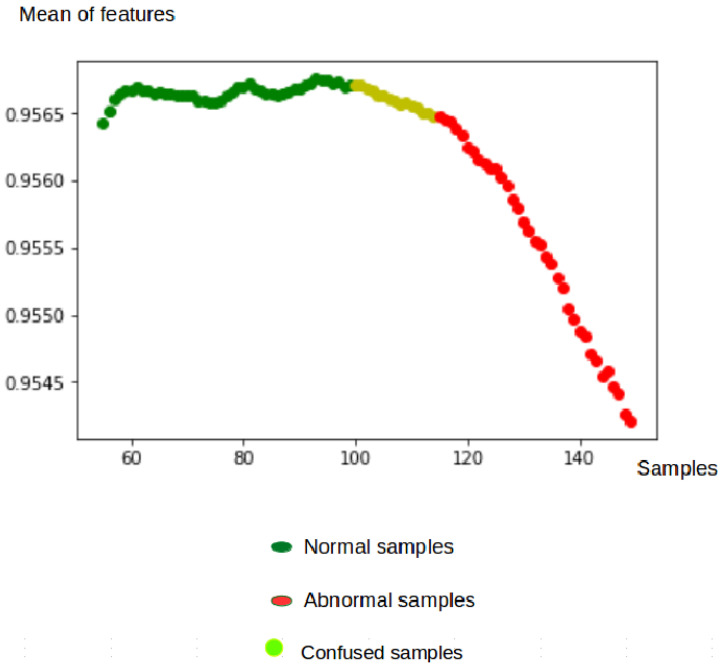
Mean of features at the testing phase.

Figure 10 shows that the characteristics of the normal samples have an average very close to 1, but those of the abnormal samples are less close to 1. The confused samples are obtained when the anomalies start to appear. This illustrates the capacity of the algorithm to detect the abnormal events in a timely manner.

From the presented results, we can see that our architecture is able to separate normal events from abnormal events. This is due to the specificity of our architecture, which is the ability to automatically extract deep features and contextual information from input frames that correctly express the difference between normal and abnormal events.

## 5. Conclusions

In this paper, we propose a new, unsupervised learning method based on deep end-to-end architecture for the detection of anomalies in UAV video streams. The main advantage of this method is its efficiency to jointly extract the optical flow features and to integrate a compactness regularization term during training. This method proves promising in terms of detection and localization of anomalies by UAV cameras and gives very high performance experimental results compared to state-of-the-art methods. Our future work is to study these results by setting up an on-board computer on the UAV for real-time anomaly detection application.

## Figures and Tables

**Table 1:** Our architecture hyperparameters.

Layer	Filters	Kernel (h,w,d)	Stride (h,w,d)
Conv1	64	[11,11,1]	[2,2,1]
Conv2	128	[3,3,1]	[1,1,1]
Conv3	256	[3,3,3]	[2,2,1]
Conv4	512	[3,3,1]	[2,2,1]
Conv5	64	[11,11,1]	[2,2,1]
Conv6	128	[3,3,1]	[1,1,1]
Conv7	256	[3,3,3]	[2,2,1]
Conv8	512	[3,3,1]	[2,2,1]
Concat	1024	––	––
Deconv1	512	[3,3,1]	[2,2,1]
Deconv2	256	[3,3,3]	[2,2,1]
Deconv3	128	[3,3,1]	[1,1,1]
Deconv4	1	[11,11,1]	[2,2,1]
Deconv5	512	[3,3,1]	[2,2,1]
Deconv6	256	[3,3,3]	[2,2,1]
Deconv7	128	[3,3,1]	[1,1,1]
Deconv8	1	[11,11,1]	[2,2,1]

**Table 2:** EER and AUC for frame-level comparisons on MDVD.

Methods	EER	AUC
VGG+LSTM [5]	–	72.75
VGG [5]	–	50.12
Ours	19.85	85.3

**Table 3:** EER and AUC for frame-level comparisons on the Ped2 dataset.

Methods	EER	AUC
Mehran. [34]	40	-
Kim. [35]	30.71	-
PCA [36]	29.20	73.98
CAE(FR) [37]	26.00	81.4
S. Hamdi [38]	14.50	-
Sabokrou [39]	8.2	-
ours	8.1	94.9

**Table 4:** Compactness loss importance.

	EER	AUC
our (without compactness)	23	78.2
our (with compactness)	19.85	85.3

## Data Availability

The study did not report any data.
